# Effects of *sang-qi* granules on blood pressure and endothelial dysfunction in stage I or II hypertension: study protocol for a randomized double-blind double-simulation controlled trial

**DOI:** 10.1186/s13063-019-3690-z

**Published:** 2020-01-06

**Authors:** Haoyue Shi, Deshuang Yang, Jiajun Qiao, Rui Sun, Ruihan Li, Chunlin Zhu, Ruiqing Jing, Liping Liu, Li Huang, Lin Li

**Affiliations:** 1 Beijing Hospital of Traditional Chinese Medicine, Capital Medical University, Dong Cheng District, Beijing, 100010 China; 2 Graduate School, Beijing University of Chinese Medicine, Chao Yang District, Beijing, 100029 China; 3 Department of Integrative Cardiology,, China-Japan Friendship Hospital,, Chao Yang District, Beijing, 100029 China

**Keywords:** Hypertension, Endothelial dysfunction, Traditional Chinese medicine (TCM), *Sang-qi* granules

## Abstract

**Background:**

Worldwide, hypertension is an important public health challenge because of its high prevalence and the concomitant risks of cardiovascular disease. It induces half of the coronary heart disease and approximately two-thirds of the cerebrovascular disease burden. Vascular endothelial dysfunction has important roles in the pathophysiology of essential hypertension. Types I and II hypertension can be treated with *sang-qi* granules (SQG), a Chinese herbal formula. Several experimental studies on animals have shown that SQG can lower blood pressure and myocardial fibrosis by suppressing inflammatory responses. However, no standard clinical trial has confirmed this. Whether SQG can improve endothelial cell function is unknown.

**Methods/design:**

In this randomized double-blind double-simulation controlled trial, 300 patients with stage I or II hypertension will be recruited and randomly allocated in a 1:1:1 ratio to group A (treatment with SQG and placebo instead of Losartan), group B (treatment with Losartan and placebo instead of SQG), and group C (treatment with SQG and Losartan). In this study, 10 g of SQG (or its placebo) will be administrated twice a day and 50 mg of Losartan (or its placebo) will be administrated once in the morning. The primary endpoint is the drug efficiency for each of the three groups. The secondary endpoints are the change in average systolic and diastolic blood pressure during the day and the night, the change in the rate at which blood pressure drops at night, assessment of target organ damage (heart rate variability, ankle–brachial pressure index, and pulse wave velocity), assessment of any improvement in symptoms (Hypertension Symptom Scale, syndrome integral scale in traditional Chinese medicine, Pittsburgh Sleep Quality Index Scale, Self-Rating Anxiety Scale, Self-Rating Depression Scale, and the 36-Item Short Form Health Survey), blood lipids, serum indicators of vascular function (changes in serum levels of ET-1, TXA2, NO, and PGI2), and safety indicators.

**Discussion:**

This study aims to provide clinical evidence on the efficacy and safety of SQG in the treatment of hypertension. Moreover, the possible mechanism by which SQG may lower blood pressure will be explored by observing the protective effect of SQG on vascular endothelial function, as well as its effect on related clinical symptoms, risk factors, and the target organs of hypertension.

**Trial registration:**

Chinese Clinical Trials Registry, ChiCTR1800016427. Registered on 1 June 2018.

**Electronic supplementary material:**

The online version of this article (10.1186/s13063-019-3690-z) contains supplementary material, which is available to authorized users.

## Background

Hypertension, a major cause of cardiovascular disease, has become a leading global health challenge. Meta-analyses of observational studies show that hypertension is associated with an increased risk of cardiovascular disease, end-stage renal disease, subclinical atherosclerosis, and all-cause mortality [1]. Although there are very beneficial strategies for treating hypertension, such as evidence-based methods for alleviating risk factors and use of angiotensin-converting enzyme inhibitors, β-blockers, etc., outcomes could be better, since hypertension is often not completely controlled. Data for 38,276 adults who participated in the National Health and Nutrition Examination Survey demonstrate that the age-standardized prevalence of hypertension decreased from 48.4% in 1999–2000 to 45.4% in 2015–2016. However, the absolute burden of hypertension consistently increased, from 87.0 million in 1999–2000 to 108.2 million in 2015–2016 [2]. In addition, more than one-fourth of Chinese adults have hypertension and its prevalence in China has increased significantly in recent decades [3].

As a complex condition, the precise cause of essential hypertension is unknown [4]. One potential mechanism is endothelial dysfunction. The endothelium plays a vital role in regulating vascular tone, cell growth, and the interaction between leukocytes, thrombocytes, and the walls of blood vessels [5]. Endothelial cells can produce a variety of substances critical for the maintenance of vascular homeostasis, with nitric oxide (NO) being the most important [6]. When NO is released from endothelial cells, it relaxes the smooth muscle, which results in vasodilation. Many studies have suggested that endothelial dysfunction is an early event in the pathophysiology of essential hypertension. It may contribute to subclinical target organ damage and the progression of atherosclerosis [7]. Thus, endothelial dysfunction has emerged as a promising therapeutic target for treating hypertension.

**Fig. 1:**
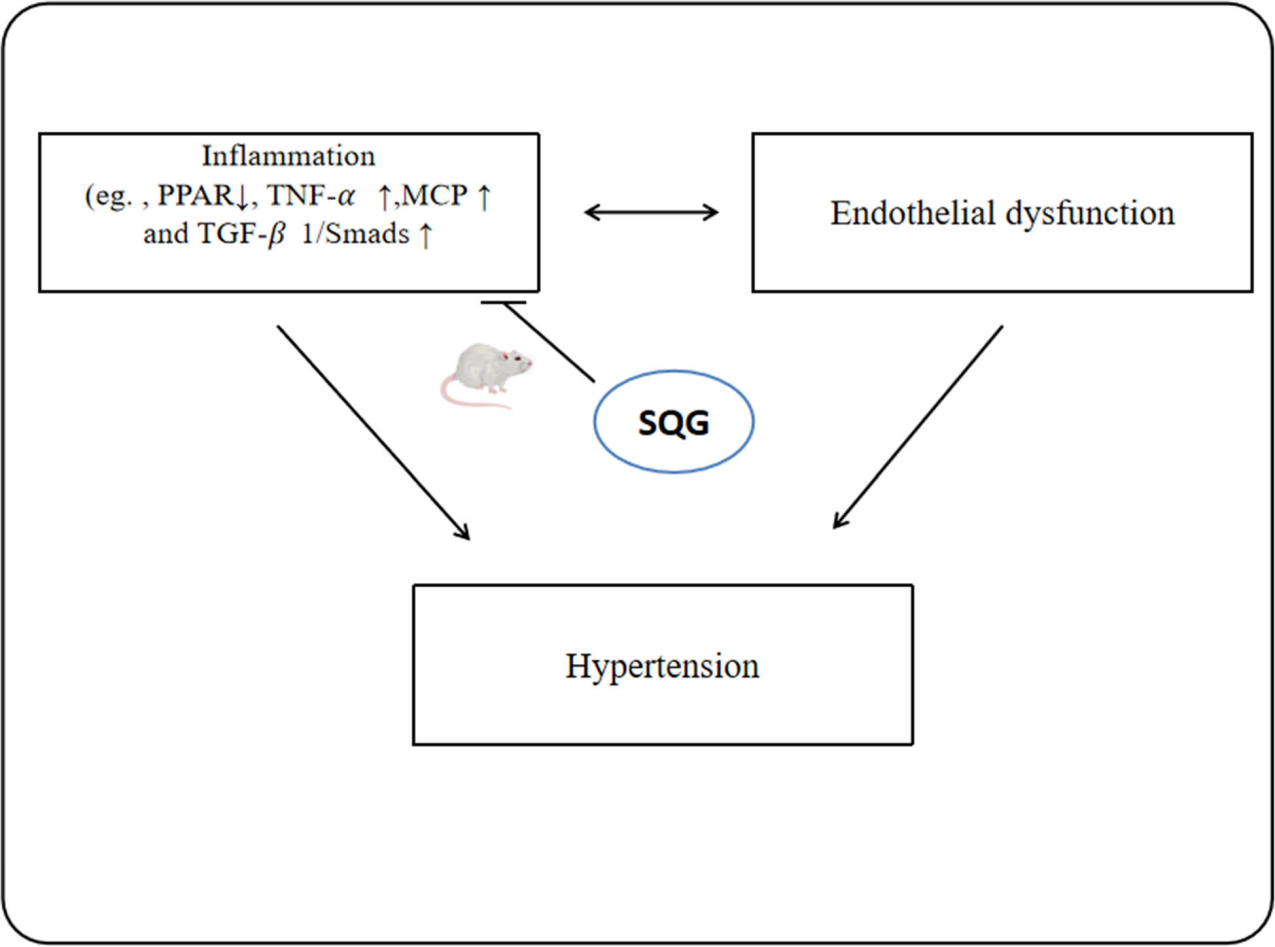
Schematic illustrating the relationship among inflammation, endothelial dysfunction, and hypertension. SQG may reduce the blood pressure and improve vascular endothelial function due to its anti-inflammatory properties. SQG *sang-qi* granules

An early mention of hypertension occurs in the *Huangdi Neijing*, a classic work on traditional Chinese medicine (TCM). Hypertension was classified as headaches and vertigo in ancient China. At that time, traditional Chinese herbal medicine was used to treat the disease, but there was no medical research looking for evidence of efficacy. *Sang-qi* granules (SQG) comprise traditional Chinese herbs used to treat hypertension. Its main ingredients are *Loranthus parasiticus* (*sangjisheng*), *Lycium barbarum* L. t (*gouqizi*), *CassiatoraL tora* Linn. (*juemingzi*), *Dendranthema indicum* (*yejuhua*), and *Salvia miltiorrhiza* Bunge (*danshen*). Our team has conducted a nano liquid chromatography–mass spectrometry analysis of SQG to evaluate its chemical components. It mainly contains betaine, chlorogenic acid, rutin, montfermanin, hesperidin, tanshinone, and quercetin. Previous animal studies by us show that SQG can lower blood pressure and myocardial fibrosis by suppressing inflammatory responses, such as upregulating the expression of PPAR and downregulating the expression of TNF-α, MCP-1, and TGF-β1/Smads, which are signaling molecules involved in myocardial fibrosis pathogenesis in cardiomyocytes [8]. Studies have confirmed that inflammation leads to the development of hypertension and is also a key factor in endothelial dysfunction [9]. Therefore, SQG may be able to regulate vascular endothelial function and may lower blood pressure by inhibiting the inflammatory response (Fig. 1). Consequently, we designed this clinical study to assess the efficacy and safety of SQG on treating endothelial dysfunction in hypertension.

## Methods/design

### Objectives

Our study aims to assess the clinical effect of SQG on vascular endothelial dysfunction in patients with stage I or II hypertension, to provide a reliable experimental basis for the early prevention and treatment of target organ damage in hypertension, and to observe whether TCM plus Western medicine has a better curative effect than Western medicine alone.

### Design

This study is a randomized double-blind double-simulation controlled trial. Participants will be recruited from the China–Japan Friendship Hospital through notices at the hospital and newspaper advertisements.

**Fig. 2:**
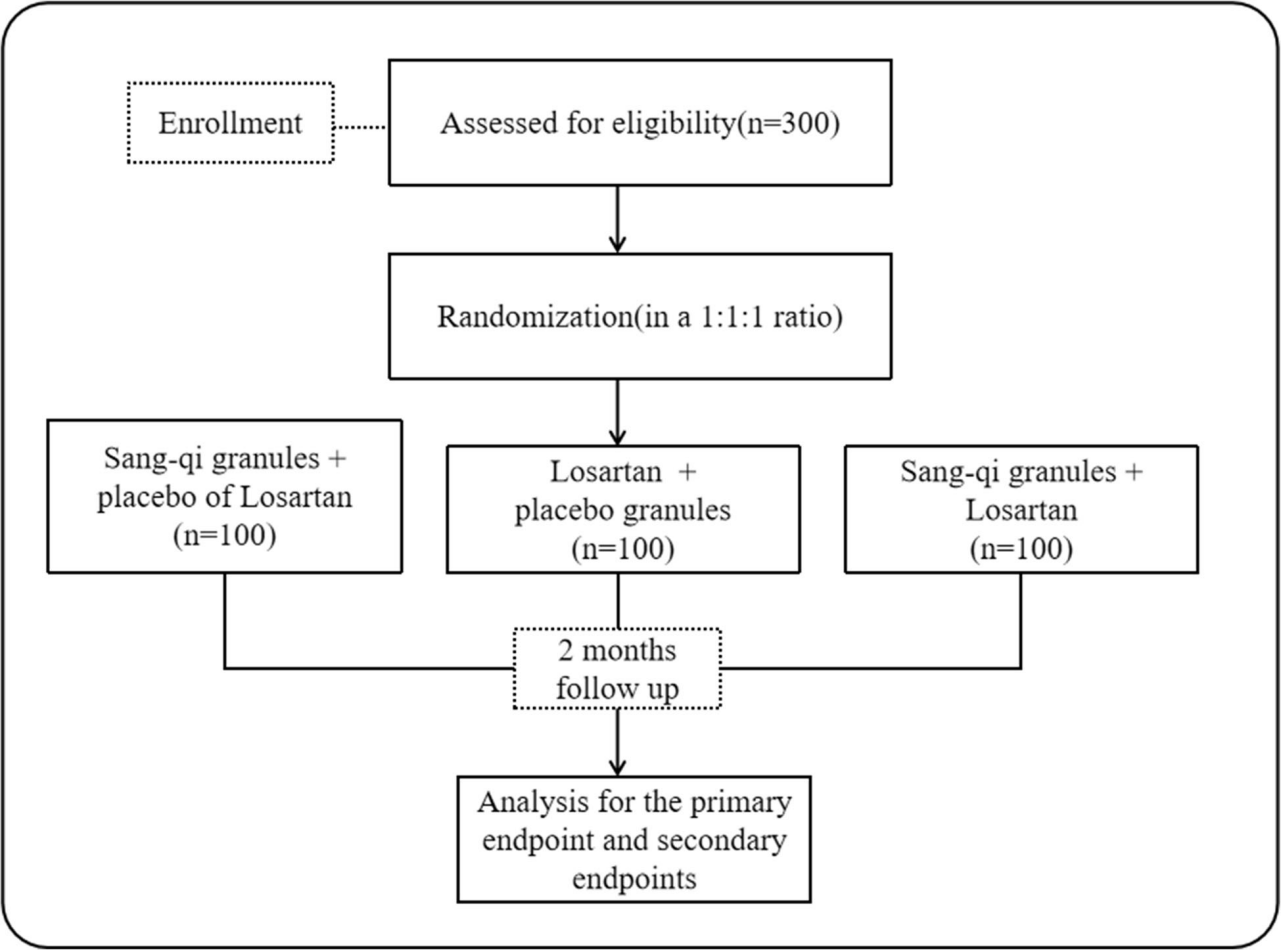
Flowchart of this study

This clinical trial will recruit patients with stage I or II hypertension, and it will determine the efficacy and safety of SQG. All examinations and tests will be carried out according to the clinical trial plan. Participants will be randomized before the first treatment. Altogether, 300 subjects with stage I or II hypertension will be randomly assigned to three groups in a 1:1:1 ratio: group A (treatment with SQG and placebo instead of Losartan), group B (treatment with Losartan and placebo instead of SQG), and group C (treatment with SQG and Losartan) (Fig. 2). Recruited patients will receive an 8-week treatment (10 g of SQG or the placebo for SQG will be given twice a day in the morning and evening, and 50 mg of Losartan or the placebo for Losartan will be given every morning). SQG and its placebo are produced in China in accordance with the quality standards prescribed in the *Chinese Pharmacopoeia* http://wp.chp.org.cn/front/chpint/en/. Upon completion of the 8-week treatment, follow-up tests will be conducted at weeks 2, 4, 6, and 8 after randomization.

### Recruitment

**Fig. 3:**
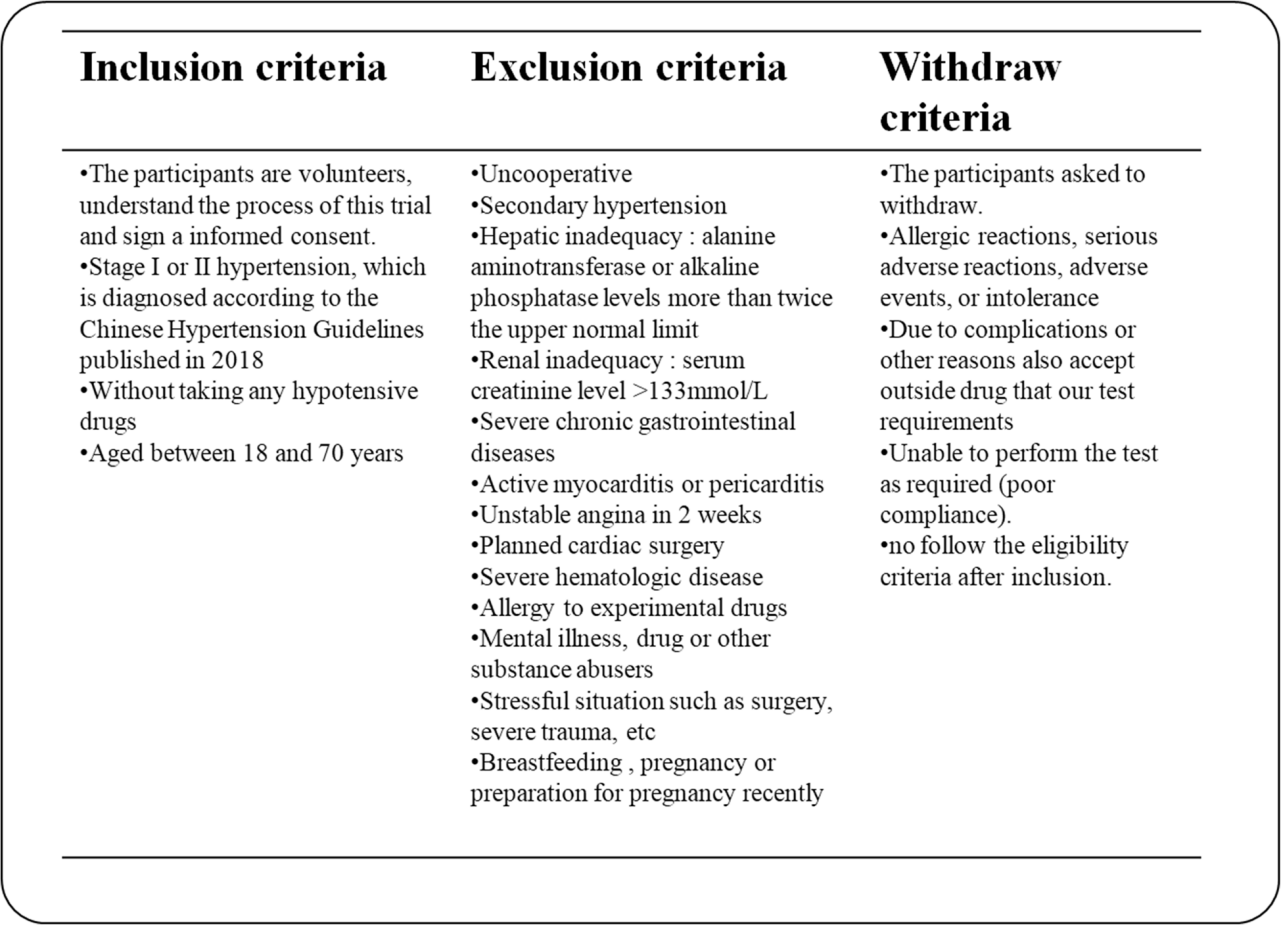
Inclusion, exclusion, and withdrawal criteria

Patients with hypertension, recruited in the relevant clinic and ward of the China–Japan Friendship Hospital through advertisements and referrals, will be screened. The principal investigator, together with a well-trained attending physician, will identify potentially eligible patients based on the eligibility criteria. Then, a researcher will inform the patient face to face of the whole schedule, including the aim, procedures, possible side effects, and benefits of the study and SQG. If a patient agrees to take part in the study, they must voluntarily sign the informed consent form before randomization. The inclusion, exclusion, and withdrawal criteria are listed in Fig. 3.

### Ethics

The protocol (version 1.0, dated 30 March 2018) was approved by the clinical research ethics committee of the China–Japan Friendship Hospital (approval 2018–59-K43), where the study will take place. It has been registered with the Chinese Clinical Trial Registry (ChiCTR1800016427), which is listed in the WHO Registry Network. The trial complies with the principles of the Declaration of Helsinki and the principles of good clinical practice [10, 11]. Each participant must sign an informed consent form before enrollment, and they have right to withdraw from the trial at any time. Additional file 1 is the populated SPIRIT checklist.

### Randomization and blinding

Randomization Biostatisticians used Statistical Analysis System (SAS, Version 9.4) to generate random tables. All the selected patients were included in the clinical trial according to the order of random table numbers. The ratio of three groups was1:1:1.

Blinding the allocation code for each participant was placed in one of 300 sealed opaque envelopes. The envelopes also contained details of the treatment options, possible adverse events, and emergency measures. The envelopes were not accessible to the researchers, participants, clinical trial pharmacists, data managers, or statisticians, none of whom know the location of the envelopes nor the treatment plan for any participants. The drug was similar to its placebo in each group. The manufacturer labeled the random codes on the package according to the principles of GCP.

### Intervention

SQG (production batch number 181201), the placebo for SQG (production batch number 181201), and the placebo for Losartan (production batch number 181001) were produced and packed in a single batch by Jiangxi Puzheng Pharmaceutical Co., Ltd. (Unified Social Credit Identifier 91360823744294486X). As tested, the drug conformed with the quality specified in the Chinese medicine standards published by the State Food and Drug Administration. The batch of SQG was made from 200 kg of original drug flow paste and 180 kg of dextrin. The batch of the placebo for SQG was made from 5 kg of caramel, 120 kg of dextrin, and 25 kg of original drug flow paste. The main component of the placebo for Losartan is dextrin. Losartan was bought from Pharmaceutical Branch of China Pharmaceutical Holdings Beijing Co., Ltd. (Unified Social Credit Identifier 91110101101297579G).

### Endpoint measurements

#### Primary endpoint

The primary endpoint is the difference in drug efficiency between the three groups. Efficiency is defined as:

1. Blood pressure is reduced to a threshold set out in *Guiding Principles for Clinical Research of New Chinese Medicines* [12].
2. Flow-mediated dilation has increased by at least 2% [13].

#### Secondary endpoints

1. The change in average systolic and diastolic blood pressure during the day and the night, as assessed by 24-h blood pressure monitoring.
2. The change in the rate at which blood pressure drops rate at night, as assessed by 24-h blood pressure monitoring.
3. Assessment of target organ damage, using:
  - Heart rate variability, based on routine 24-h Holter recordings, which provide a sensitive and noninvasive measurement of autonomic input to the heart [14]
  - The ankle-brachial index (ABI), which is the ratio of the systolic blood pressure (SBP) measured at the ankle to that measured at the brachial [15].
  - Pulse wave velocity, which is the velocity at which the blood pressure pulse propagates through the circulatory system. It is calculated as the distance between the two arterial sites divided by the time delay for a pulse at the two sites [16].
4. Assessment of any improvement in symptoms, using:
  - Hypertension Symptom Scale (Additional file 2)
  - TCM Syndrome Integral Scale (Additional file 3)
  - Pittsburgh Sleep Quality Index Scale [17, 18]
  - Self-Rating Anxiety Scale [19]
  - Self-rating Depression Scale [20]
  - 36-Item Short Form Health Survey, which assesses quality of life [21, 22]
5. Blood lipids: Changes in levels of total cholesterol, triglycerides, low-density lipoproteins, and high-density lipoprotein will be assessed at baseline and treatment endpoint
6. Serum indicators of vascular function: Changes in serum levels of endothelin 1 (ET-1), thromboxane A2 (TXA2), NO, and prostaglandin I2 (PGI2) will be assessed at baseline and treatment endpoint
7. Safety indicators: Levels of creatinine, blood glucose, homocysteine, and uric acid, and routine blood and urine test will be assessed at baseline and treatment endpoint

### Laboratory tests

Levels of creatinine, blood glucose, blood lipids, homocysteine, and uric acid, plus routine blood and urine tests will be assessed at the Clinical Laboratory, China–Japan Friendship Hospital Beijing, China. Creatinine, blood glucose, blood lipids, homocysteine, uric acid, angiotensin II, and high-sensitivity C-reactive protein will be tested by an analyzer (Beckman Coulter Chemistry Analyzer AU5800, Beckman Kurt Co., Ltd., US). Blood will be tested by a blood analyzer (Sysmex Blood Routine Analyzer Xn90, SYSMEX Co., Ltd., Japan) and urine will be tested by a fully automated urine analyzer (AUTION MAX AX-4030, Aikolai Co., Ltd., Japan).

Blood samples will be sent to the Clinical Research Center of the China–Japan Friendship Hospital (transported at 2–6 °C) within 10 min of collection. After the blood is centrifuged, the supernatant fluid will be pipetted, stored in a cryotube, and placed in a freezer at −80 °C. The cryopreserved supernatant fluid will be tested using an enzyme-linked immunosorbent assay (ELISA) kit or chemical kit within 6 months to assess serum levels of ET-1, TXA2, and PGI2. Serum NO levels will be measured by the nitrate reductase method using a nitric oxide assay kit. The experimental procedure will be carried out by a professional technician following the manufacturers’ instructions. Instruments used during testing include an electronic balance (YP30001, Shanghai Guangzheng Medical Instruments Co., Ltd., China), a mini-shaker (MS1 Minishaker, IKA (Guangzhou) Instruments and Equipment Co., Ltd., China), a desktop low-speed automatic-balancing centrifuge (LDZ5–2, Beijing Jingli Centrifuge Co., Ltd., China), an electric constant-temperature water tank (HW. W21.600, Beijing Changfeng Instrument and Meter Company, China), and a multi-function microplate reader (SpectraMax M2, Meigu Molecular (Shanghai) Instruments Co., Ltd., China).

### Data collection and management

**Fig. 4:**
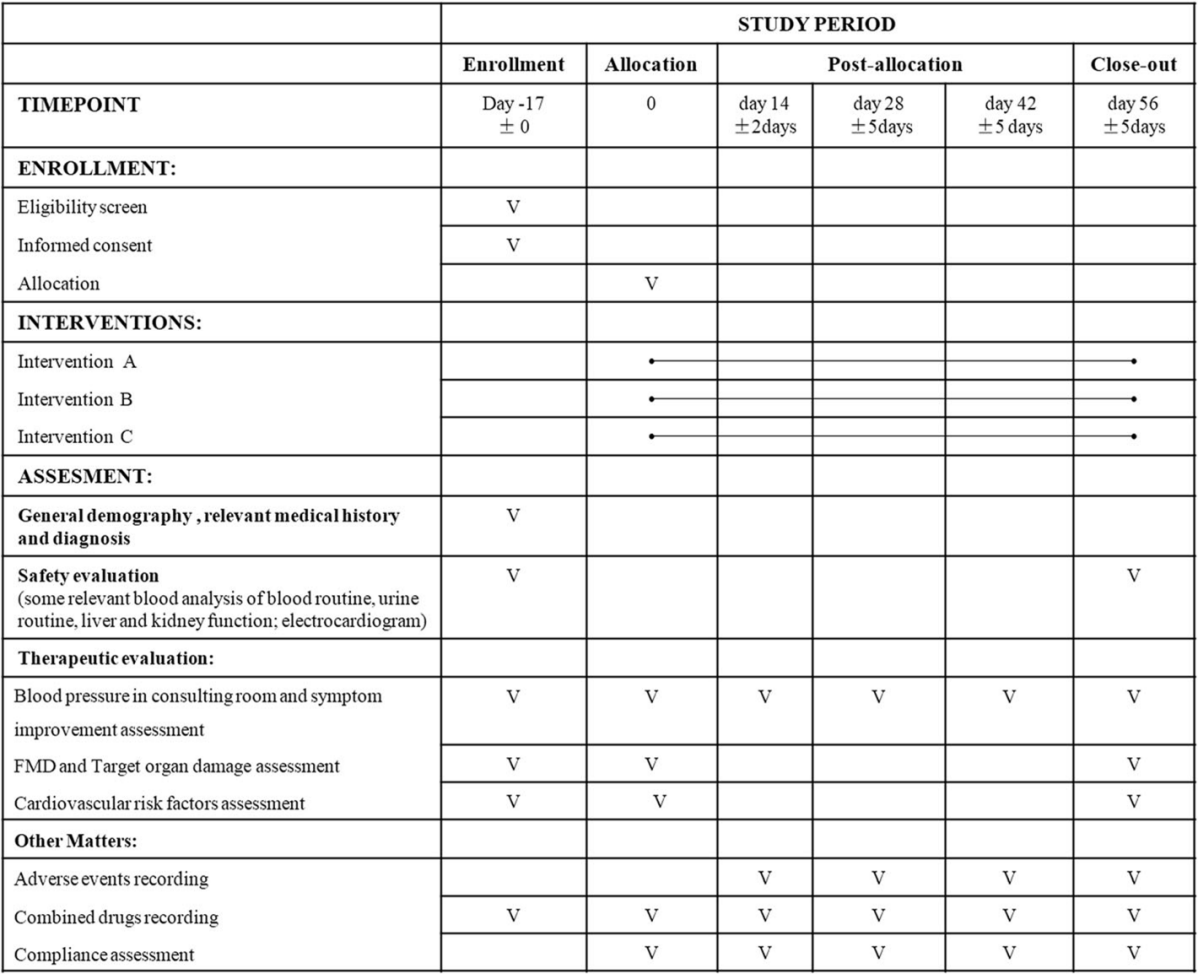
Schedule of enrolment, interventions, and assessments

Data for each participant will be recorded in a case report form. In addition to the enrollment evaluation (−17 ± 0 days), each participant will attend an evaluation visit when allocated and every 14 days afterwards during the trial (0 days, 14 ± 2 days, 28 ± 5 days, 42 ± 5 days, and 56 ± 5 days). Each evaluation will include a physical examination, an assessment of any improvement in symptoms, an assessment of compliance with medications, questions about other medications, and questions about adverse events. Blood samples and 24-h blood pressure monitoring data will be collected only at the enrollment and close-out visits. Fig. 4 shows the schedule of enrolment, interventions, and assessments.

Data will be input into a clinical data management system through a website (http://www.cardiar.com/zryygxy) by two research staff. The system, which will be protected by passwords, will be managed by Beijing Cardiar Technology Co., Ltd. (Unified Social Credit Identifier 91110 10,655 68,170 240). All data supporting the conclusions of this trial will be stored in this system. To ensure data integrity and ease of storage, we will use data rules, valid values, and scope checks. The system will report missing data and data errors. The data may be changed but all changes will be tracked. The conduct of the trial will be audited by Beijing Inruida Pharmaceutical Technology Co., Ltd. (Unified Social Credit Identifier 91110105MA00H1U54L). These data management procedures will be undertaken completely independently of the investigators and the sponsor. All personnel involved in data entry and data management will sign a confidentiality agreement to prevent data leakage. The personal information of all participants will be fully protected. The original case report forms will be kept for 5 years after the end of the trial.

### Adverse events

Adverse events are defined as an accident, any signs of discomfort, or any disease symptoms, such as hypertensive emergencies, bleeding, hematoma, syncope, severe pain, and local infection. All details of adverse events that occur during the observation period will be recorded on the participant’s case report form. The clinical researchers must report each adverse event to the research leader, sponsor, and the ethics committee within 24 h. The ethics committee will recommend relevant treatment.

### Statistical analysis

#### Sample size calculation

The trial will check for superiority between group B (treatment with Losartan and placebo instead of SQG) and group C (treatment with SQG and Losartan), and for non-inferiority between group A (treatment with SQG and placebo instead of Losartan) and group B (treatment with Losartan and placebo instead of SQG).

The sample size was estimated based on clinical research literature and preliminary clinical data on blood pressure and FMD for hypertensive subjects. Given a type-I error rate of α=0.025 and a type-II error rate of β=0.1. The efficiency of the group C was estimated as 90%, and the group B as 70%. Superior efficiency cutoff is 10%. The calculation result was 78 patients in group B and group C by PASS 11 software. After considering the expulsion rate and the requirement of minimum case number of the GCP (A randomized controlled clinical trial requires at least 100 patients each group). We estimated 100 patients in group B and group C. Group A was an exploratory trail with the above two groups, 100 patients initially identified. Above all, we decided to recruit 300 patients during this trail.

### Data analysis

Data entry and data management will be undertaken by an independent data administrator to ensure data accuracy. A professional statistician will perform the data analysis. We will use the intention-to-treat principle to analyze the efficacy and safety of SQG. For continuous variables, an independent two-sample Student’s *t* test will be used for comparisons between the two study groups, and a paired test will be used for intra-group comparisons. A χ^2^ test will be used for categorical variables. For continuous data with a normal distribution, a Wilcoxon test will be used. *P* < 0.05 is considered to be statistically significant, and all tests are two-tailed.

## Discussion

Hypertension is an important risk factor for cardiovascular disease [23]. However, data from a recent national survey in the United States found that about one-half of all hypertensive adult patients had uncontrolled blood pressure [24], which may contribute to the widespread morbidity and mortality reported globally [25]. Therefore, better treatment methods are required, which was the motivation to consider SQG in this research. According to previous studies, SQG can promote the expression of the PPAR pathway and inhibit the expression of the TNF-α, MCP-1, and TGF-β1/Smads signaling molecules, which would suppress inflammatory responses. Furthermore, SQG may lower blood pressure and protect the target organs of hypertension [8]. Vascular endothelial dysfunction is also a factor in the development of hypertension, since it may contribute to increased systemic vascular resistance. Inflammation is a potential mechanism of endothelial dysfunction. For example, ligand-activated PPAR-γ decreases the inflammatory responses in cardiovascular cells, particularly in endothelial cells [26]. TNF has been proven to attenuate NO production by destabilizing eNOS mRNA and to restore endothelial-dependent vasodilation [27]. However, there is a lack of high-quality clinical research on the supposed benefits of SQG. Thus, we designed this trial to determine the efficacy and safety of SQG in the treatment of hypertension.

To ensure the reliability of our conclusions, the experimental design and study implementation are conducted with strict quality control. Statisticians were involved in estimating the sample size, generating the random allocation sequence, randomization concealment, and blinding. A training session was held to explain the study protocol and to clarify the standard operating procedures. Specialized laboratories will process the biochemical samples. Additionally, the progress and quality of the trial will be monitored by a clinical research organization.

It is hoped that this trial will provide high-quality evidence on the efficacy and safety of SQG in treating hypertension, thus supporting the clinical application of SQG. Additionally, the progress and quality of the trial will be monitored by a clinical research organization of China-Japanese Friendship Hospital.

### Trial status

This protocol (version 1.0 date 30 March 2018) was approved by the clinical research ethics committee of the China–Japan Friendship Hospital (approval 2018–59-K43), Beijing, China, in May 2018. The study started on 13 February 2019. Currently, 20 patients have been recruited. The study will be finished by December 2020.

## Additional files

Additional file 1: SPIRIT checklist.

Additional file 2: Hypertension Symptom Scale.

Additional file 3: TCM Syndrome Integral Scale.

## Data Availability

Not applicable.

## Abbreviations

ELISA: Enzyme-linked immunosorbent assay
ET-1: Endothelin 1
FMD: Flow-mediated dilation
PGI2: Prostaglandin I2
SQG: *Sang-qi* granules
TCM: Traditional Chinese medicine
TXA2: Thromboxane A2
