# Supplementary material for: Effects of sang-qi granules on blood pressure and endothelial dysfunction in stage I or II hypertension: study protocol for a randomized double-blind double-simulation controlled trial
Source: Trials. 2020 Jan 6;21:12. doi: 10.1186/s13063-019-3690-z (PMC6945446; doi:10.1186/s13063-019-3690-z)
Supplement: Supplementary file 2 [file 13063_2019_3690_MOESM2_ESM.docx]

Hypertension symptom scale

1. The following questions ask how much your hypertension symptom affected your life during the past two weeks.
2. After each question, circle the 0, 1, 2, 3, or 4 to show how much you feel these days.

Item	Zero point	One point	Two points	Three points	Four points
headache or heavy-head	none	occasionally	sometimes	often	always
vertigo or tinnitus	none	occasionally	sometimes	often	always
palpitation	none	occasionally	sometimes	often	always
shortness of breath	none	occasionally	sometimes	often	always
chest pain	none	occasionally	sometimes	often	always
dizziness	none	occasionally	sometimes	often	always
edema	none	occasionally	sometimes	often	always
weakness	none	occasionally	sometimes	often	always
diuresis	none	occasionally	sometimes	often	always
stiff neck or shoulder	none	occasionally	sometimes	often	always
