# Supplementary material for: Effects of sang-qi granules on blood pressure and endothelial dysfunction in stage I or II hypertension: study protocol for a randomized double-blind double-simulation controlled trial
Source: Trials. 2020 Jan 6;21:12. doi: 10.1186/s13063-019-3690-z (PMC6945446; doi:10.1186/s13063-019-3690-z)
Supplement: Supplementary file 3 [file 13063_2019_3690_MOESM3_ESM.docx]

TCM syndrome integral scale

1. The following questions are used to evaluate the symptoms.
2. Please score according to the change of symptoms in these two weeks.

main symptoms	0 point	2 points	4 points	6 points
dizziness	none	occurs randomly	sight rotation, can't walk	dizzy, can't stand
headache	none	occurs randomly	continuous headache; tolerable	unbearable severe headache
sore waist and weak knees	none	occurs occasionally; after exhaustion, does not affect Work and life	slightly, can be relieved after rest, can work normally	symptoms occur, highly noticeable, it will still appear at rest
chest distress	none	slightly	obviously, sigh occasionally	chest distress; hard to breathe
heavy-head	none	occasionally	frequently	always
irritable	none	occasionally	frequently	always
feverish sensation over the five centers (palms, soles, and chest)	none	palms and feet heat, dysphoria in chest occasionally	palms and feet heat and wanted to be undressed, dysphoria in chest frequently	palms and feet are hot, want to hold cold objects, dysphoria in chest all day long
vomiting; phlegm and salivation	none	occasionally	frequently	always
intolerance of coldness; cold body	none	slightly	obviously	intolerance of coldness; cold body desire of adding cloths

secondary symptoms	0 point	1 point	2 points	3 points
red complexion	none	occasionally	frequently	always
inflamed eyes	none	occasionally	frequently	always
dry mouth	none	slightly	frequently	desire of water

constipation	none	dry stool, once a day	constipation, once every two days	the stooling is difficult, once a few days
diarrhea	none	stool is thin, 1~3 times a day	stools or watery diarrhea, 3 to 5 times a day	diarrhea more than 5 times a day
concentrated and dark urine	none	slightly yellow urine	yellow scanty urine	dark scanty urine, difficulty in micturition
physically impaired	none	Body feels heavy, but do not have any affection to activities.	heavy limbs, activities laborious	the limbs are heavy and wrap, and the activity is difficult
sticky in the mouth	none	slightly sticky, does not affect appetite	sticky in the mouth, loss of appetite	sticky and uncomfortable in the mouth, do not want to eat at all
bitter taste	none	bitterness in the mouth at morning time	The mouth is too bitter to taste.	bitter and dry
tastelessness	none	slightly tasteless in the mouth	tasteless in the mouth	tastelessness and don't want to eat
cardiopalmus	none	palms and feet fever, dysphoria in chest occasionally	palms and feet fever and wanted to be undressed, dysphoria in chest frequently	palms and feet are hot, want to hold cold objects, dysphoria in chest all day long
shortness of breath	none	post-activity shortness of breath	short breath at rest	always

insomnia	none	a slight decrease in sleep	insomnia frequently	can't fall asleep
tinnitus	none	Tinnitus is slight, occasionally appears, lasts for a few seconds, does not affect hearing	Tinnitus is heavier, often occurs, lasts for a few minutes, affects hearing slightly	Tinnitus is like a cicada and a train, sustained, obviously affecting work and sleep
amnesia	none	occasionally forget things, still remember	common things easy forgettable; hard to remember things	can't remember things and can't recall things
anorexia	none	poor appetite, appetite reducing, food intake is reduced by no more than 1/4	loss of appetite, appetite reducing, food consumption is reduced by 1/4~1/2	poor appetite, no hunger, reduced food intake by more than 1/2
sweating	none	occasionally	frequently	always

dry eyes		none	Eyes are slightly dry, easily get tired when staring at things	Eyes are dry and target objects are often blurred.	Eyes are dry, target objects are dark and unclear
high frequency of enuresis nocturia		none	nocturia once	nocturnal urine two to three times at night	nocturia more than three times at night
burnout		none	occasionally feels slightly tired, labor-resistant, can stick to slight physical labor.	feeling weak after normal activities, occurs occasionally; can lightly support daily activities.	still tired after rest, symptom appears all day, cannot support daily life
edema		none	morning eyelid edema, swollen foot in the afternoon, the swelling is faintly visible	eyelids and lower extremity edema, the edema is slight concavity	whole body edema, and the edema is obviously concavity
**tongue and pulse**	tongue	□normal □dim □blush □red □light red<br>□light □purple dark □freckle<br>□thin □fat □old □soft □tooth mark<br>□dry □less of fluid □wet			
	tongue fur	□normal □black □sallow □yellow □white<br>□thin white □sticky □few coating on the tongue			
	pulse	□normal □sedimentation □floating □weak<br>□string □slip □tight<br>□soaking □fineness □uneven □bradycardia □rapid<br>□slow pulse with irregular intervals □intermittent			

Total score:
